# Unique metabolism of different glucosinolates in larvae and adults of a leaf beetle specialised on Brassicaceae

**DOI:** 10.1038/s41598-022-14636-6

**Published:** 2022-06-28

**Authors:** Jeanne Friedrichs, Rabea Schweiger, Caroline Müller

**Affiliations:** Department of Chemical Ecology, Bielefeld University, Universitätsstr. 25, 33615 Bielefeld, Germany

**Keywords:** Biochemistry, Ecology, Evolution

## Abstract

Brassicaceae plants contain glucosinolates, which are hydrolysed by myrosinases to toxic products such as isothiocyanates and nitriles, acting as defences. Herbivores have evolved various detoxification strategies, which are reviewed here. Larvae of *Phaedon cochleariae* (Coleoptera: Chrysomelidae) metabolise hydrolysis products of benzenic glucosinolates by conjugation with aspartic acid. In this study, we investigated whether *P. cochleariae* uses the same metabolic pathway for structurally different glucosinolates, whether the metabolism differs between adults and larvae and which hydrolysis products are formed as intermediates. Feeding experiments were performed with leaves of watercress (*Nasturtium officinale*, Brassicaceae) and pea (*Pisum sativum*, non-Brassicaceae), to which glucosinolates with structurally different side chains (benzenic, indole or aliphatic) or their hydrolysis products were applied. Samples were analysed by UHPLC-QTOF-MS/MS or TD–GC–MS. The same aspartic acid conjugates as previously identified in larvae were also detected as major metabolites of benzenic glucosinolates in adults. Indol-3-ylmethyl glucosinolate was mainly metabolised to *N*-(1*H*-indol-3-ylcarbonyl) glutamic acid in adults and larvae, while the metabolism of 2-propenyl glucosinolate remains unclear. The metabolism may thus proceed primarily via isothiocyanates rather than via nitriles, while the hydrolysis occurs independently of plant myrosinases. A detoxification by conjugation with these amino acids is not yet known from other Brassicaceae-feeders.

## Introduction

As sessile organisms, plants have evolved different strategies to defend themselves against herbivores, while insects which feed on those plants show various adaptations to these defence systems. For example, plants produce morphological structures or specialised metabolites and proteins with repellent, deterrent, toxic and/or anti-nutritional activities towards herbivores^1^. Several of these metabolites are non-toxic or non-active themselves, as long as they are separated from bioactivating *β*-glucosidases^2^. Such metabolites include, for example, glucosinolates, benzoxazinoids and cyanogenic glucosides^2^. While feeding, most herbivores offset the spatial separation between the glucosides and enzymes, causing the release of bioactive compounds. Therefore, herbivores have to cope somehow with the released compounds or prevent their formation. Indeed, various strategies to overcome such plant defence systems are known for herbivorous species of distinct feeding guilds^2–4^. However, it has rarely been investigated whether different development stages within species show distinct strategies, which may differ in their effectiveness (but see, e.g.,^5^).

Larvae of holometabolous insects form pupae before reaching adulthood, which goes along with drastic physiological changes. In parallel, the gut structure and microbiota community can change during metamorphosis^6^. In many species of Lepidoptera as well as in some species of Diptera, Hymenoptera and Coleoptera, larvae and adults also differ in feeding mode, for example, by switching from leaf chewing as larvae to nectar and/or pollen feeding as adults^7–9^. In contrast, larvae and adults of several Coleopteran species use the same food source. Nevertheless, the metabolism of plant-derived compounds as well as the insect’s physiology may differ at least to some degree in such species between the larval and the adult stage^10^.

Glucosinolates are found specifically in species of the Brassicales. They consist of an *S*-*β*-d-glucopyrano unit, which is connected to an *O*-sulfated (*Z*)-thiohydroximate moiety as common basic structure, but differ in their side chain^11^. Depending on the side chain structure, glucosinolates can be categorised into benzenic, indole and aliphatic glucosinolates. The structural differences in the side chains affect the formation and the biological properties of the hydrolysis products^11^. In such hydrolysis reactions, which are mostly induced when the glucosinolates and plant myrosinases come into contact, isothiocyanates, thiocyanates, nitriles, or other bioactive compounds are formed^12,13^. Thus, herbivores face the challenge of having to deal with structurally different glucosinolates as well as their distinct breakdown products and developed variable counter-adaptations to avoid poisoning by the glucosinolate-myrosinase system (^14,15^, Tables 1, S1).

**Table 1:** Overview of known metabolism strategies of glucosinolates (gls) by insects, with information about insects (species, family), mechanism and reference.

Insect		Mechanism	References
Species	Family		
*Myzus persicae*	Aphididae	Excretion of gls in the honeydew	^59^
*Brevicoryne brassicae*		High gls concentrations in insects	
*Spodoptera frugiperda*	Noctuidae	gls conjugation by glutathione S-transferase; 2-phenylethyl gls: glutathione-S-transferase activity only after induction	^60^
*Trichoplusia ni*		gls conjugation by glutathione S-transferase; no conjugation of 2-phenylethyl gls	
*Anticarsia gemmatalis*	Erebidae	gls conjugation by glutathione S-transferase; no conjugation of 2-phenylethyl gls, indol-3-ylmethyl gls	
*Athalia rosae*	Tenthredinidae	Sequestration of several gls in haemolymph, released by "easy bleeding"; no/minor sequestration of indol-3-yl-methyl gls	^20^
*Episyrphus balteatus*	Syrphidae	gls conjugation by glutathione S-transferase	^24^
*Myzus persicae* *Brevicoryne brassicae*	Aphididae	concentration of gls detected in aphid body and/or honeydew depend on host plant gls profiles	^61^
*Murgantia histrionica*	Pentatomidae	sequestration of several gls; no sequestration of 4-hydroxy-3-indolylmethyl gls and 2-phenylethyl gls	^62^
*Brevicoryne brassicae*	Aphididae	sequestration of gls, own aphid myrosinase	^63^
*Lipaphis erysimi*		accumulation of gls in small amounts	
*Plutella xylostella*	Plutellidae	desulfation of gls by sulfatase	^21^
*Pieris rapae*	Pieridae	No sequestration of gls; 4-hydroxybenzylcyanide-sulfate (putatively) present in traces	^64^
*Pieris brassicae*		No sequestration of gls; 4-hydroxybenzylcyanide-sulfate (putatively) present in traces	
*Pieris rapae*	Pieridae	formation of nitrile due to nitrile-specifier protein, excretion with faeces after further metabolism	^22^
*Myzus persicae*	Aphididae	conjugation by glutathione S-transferase	^65^
*Athalia rosae*	Tenthredinidae	sequestration of several gls in haemolymph; no myrosinase activity; 4-hydroxybenzyl gls: desulfogls present in larvae, no formation of 4-hydroxybenzylcyanide sulfate	^66^
*Anthocharis cardamines*	Pieridae	sulfation of 4-hydroxybenzylcyanide to 4-hydroxybenzylcyanide sulfate; excretion	^67^
*Pieris virginiensis*			
*Pieris napi oleracea*			
*Pieris brassicae*			
*Pieris rapae*	Pieridae	formation of nitrile due to nitrile-specifier protein, followed by conjugation with glycine; excretion	^68^
*Pieris rapae*	Pieridae	host plant-dependent metabolism: 4-hydroxybenzyl gls hydrolysed to nitrile due to nitrile-specifier protein, followed by further enzymatic steps	^69^
*Schistocerca gregaria*	Acrididae	sulfatase activity in gut, formation of desulfogls; excretion together with trace amounts of cyanide	^23^
*Brevicoryne brassicae*	Aphididae	sequestration of gls in haemolymph, embryo inside the aphid already posseses a myrosinase	^70^
*Myzus persicae*	Aphididae	conjugation of indolyl-3-methyl gls with amino acids and glutathione; excretion	^33^
*Pieris rapae*	Pieridae	Metabolism of gls through nitrile formation, hydroxylation, demethylation, sulfation, and carboxylic acid formation; excretion	^71^
*Athalia rosae*	Tenthredinidae	Sequestration of gls in larval haemolyph and adults, in larvae in higher concentrations	^72^
*Athalia liberta*			
*Athalia rosae*	Tenthredinidae	Sequestration of gls in haemolymph, metabolised to desulfo-gls and further to desulfo-gls-3-sulfate; in gut: gls metabolised to gls-3-sulfate; excretion of sulfates	^73^
*Bemisia tabaci*	Aleyrodidae	Constitutive and induced expression profiles of detoxification genes (belonging to the GSTs, P450s and COEs super families)	^74^
*Athalia* spp.	Tenthredinidae	Sequestration of gls highly species-specific	^75^
*Spodoptera littoralis*	Noctuidae	Partly conjugation with amino acids, majority unmetabolised; excretion	^17^
*Spodoptera exigua*		Varying amounts of amino acid conjugate; excretion	
*Mamestra brassicae*			
*Trichoplusia ni*			
*Helicoverpa armigera*			
*Pieris rapae*	Pieridae	Formation of nitrile (cyanide) and conjugation with glycine; detoxification by β-cyanoalanine synthase and rhodanese	^76^
*Athalia rosae*	Tenthredinidae	Rapid sequestration of gls into haemolymph	^3^
*Phyllotreta striolata*	Chrysomelidae	selective accumulation/sequestration of gls, own myrosinase	^18^
*Scaptomyza flava*	Drosophilidae	Conjugation with glutathione, subsequent Hydrolytic modification	^77^
*Scaptomyza nigrita*		Conjugation with glutathione	
*Drosophila melanogaster*			
*Brevicoryne brassicae*	Aphididae	Sequestration of certain gls particularly in nymphs, mostly excretion with honeydew in adults	^78^
*Bemisia tabaci*	Aleyrodidae	Desulfation of gls by sufatase, excretion with honeydew	^16^
*Spodoptera littoralis*	Noctuidae	Conjugation of isothiocyanates with glutathione, metabolised via mercapturic acid pathway; excretion of free isothiocyanates	^79^
*Mamestra brassicae*			
*Helicoverpa armigera*		Mostly excretion of free isothiocyanate	
*Trichoplusia ni*			
*Plutella xylostella*	Plutellidae	Desulfation of gls; excretion	
*Pieris rapae*	Pieridae	Formation of nitrile; excretion	
*Psylliodes chrysocephala*	Chrysomelidae	Sequestration of gls, desulfation and gluthatione conjugation (mercapturic acid pathway); excretion; no myrosinase activity	^19^
*Pieris rapae*	Pieridae	Activity of two rhodaneses, catalyse the transfer of sulfur from thiosulfate to cyanide	^80^
*Psylliodes chrysocephala*	Chrysomelidae	Several sulfatases with different substrate specificities	^81^
*Phyllotreta armoraciae*			
*Phyllotreta striolata*			
*Bemisia tabaci*	Aleyrodidae	Conversion of gls into glucosylated conjugates via transglucosidation; isothiocyanates and conjugates mostly excreted with honeydew	^82^
*Phyllotreta armoraciae*	Chrysomelidae	gls sequestration and own myrosinase activity	^5^
*Diadegma semiclausum*	Ichneumonidae	Conjugation of isothiocyanate with glutathione, further metabolised via mercapturic acid pathway; excretion	^83^
*Phyllotreta armoraciae*	Chrysomelidae	gls-specific sequestration and regulation of gls level by excretion	^29^
*Phaedon cochleariae*	Chrysomelidae	Amino acid conjugation with aspartic acid	^25^
*Psylliodes chrysocephala*	Chrysomelidae	Desulfation and gluthatione conjugation (mercapturic acid pathway), likely gut bacteria involved	^44^
*Bemisia tabaci*	Aleyrodidae	gls-specific desulfation; excretion with honeydew	^84^
*Phyllotreta armoraciae*	Chrysomelidae	gls sequestration, no full prevention of gls hydrolysis, inactivation of plant myrosinases in gut, ITC conjugation; excretion	^52^
*Phyllotreta armoraciae*	Chrysomelidae	gls sequestration steps: (1) uptake from gut in haemolymph, (2) transport to Malpighian tubule (MT) lumen, (3) selective uptake of gls from MT lumen into haemolymph; excretion	^85^
*Phaedon cochleariae*	Chrysomelidae	gls-specific conjugation with aspartic acid or glutamic acid	This study

More detailed information about the insects [species, family, life stage, specialist/generalist herbivore, predator and parasitoid], glucosinolates (name, trivial name, toxic product) and metabolites found in insects (name, mechanism) can be found in Supplement Table S1.

For example, some feeding generalist insect species conjugate isothiocyanates with glutathione or amino acids (^16,17^, Tables 1, S1). Some feeding specialists sequester different glucosinolates and thereby separate them from myrosinases, produce specific enzymes redirecting the glucosinolate hydrolysis to less toxic products or have their own myrosinase (^18–22^, Tables 1, S1). Only few studies investigated the detoxification of allelochemicals such as glucosinolates along the development. Upon feeding on Brassicaceae, the hemimetabolous desert locust *Schistocerca gregaria* (Orthoptera: Acrididae*)* induces a glucosinolate sulfatase in the gut, whose activity fluctuates in the larval stages and is particularly high in adult males^23^. In the holometabolous hoverfly *Episyrphus balteatus* (Diptera: Syrphidae), pupae and adults express a much higher activity of glutathione-*S*-transferases than larvae^24^. Larvae of the chrysomelid *Phyllotretra armoraciae* (Coleoptera: Chrysomelidae) show relatively low glucosinolate concentrations but a high activity of a larval myrosinase, while in adults glucosinolate concentrations are high and myrosinase activity is low^5^. Larvae of the chrysomelid *Phaedon cochleariae* metabolise intermediates derived from either isothiocyanates or nitriles produced from ingested benzenic glucosinolates to aspartic acid conjugates of aromatic acids^25^. However, it is unclear how adults of this feeding specialist metabolise the toxic hydrolysis products of benzenic glucosinolates and how both, larvae and adults, cope with structurally different glucosinolates.

In the present study, we therefore investigated the metabolism of phenylalanine- or tyrosine-derived benzenic glucosinolates (benzyl, 4-hydroxybenzyl and 2-phenylethyl as side chains) in adults of *P. cochleariae* and studied the metabolism of the tryptophan-derived indol-3-ylmethyl glucosinolate as well as the methionine-derived aliphatic 2-propenyl glucosinolate in both adults and larvae. Individuals were offered leaves of watercress (*Nasturtium officinale*, Brassicaceae) or pea (*Pisum sativum,* Fabaceae) treated with these glucosinolates or certain of their hydrolysis products. Pea lacks myrosinases and was thus used to determine whether and how glucosinolates may be metabolised independently of these enzymes. Adults, larvae and faeces as well as plant samples were tested for potential glucosinolate breakdown metabolites. We hypothesised that *P. cochleariae* metabolises the benzenic, indole and aliphatic glucosinolates in different ways, because the side chain influences the properties of the glucosinolates and their hydrolysis products. In addition, we expected that the glucosinolate metabolism differs between larvae and adults of this holometabolous species. Furthermore, we aimed to elucidate whether isothiocyanates and/or nitriles are primarily involved in the metabolism of the glucosinolates in this species.

## Results

### Glucosinolate metabolism in *P. cochleariae*

Adults and larvae of *P. cochleariae* were fed with leaf discs of watercress [dominated (~ 89% of total glucosinolate content) by the benzenic 2-phenylethyl glucosinolate, **6**; Table 2] or of pea (lacking glucosinolates) treated with one of several structurally different glucosinolates (benzenic: benzyl glucosinolate, **1**; 4-hydroxybenzyl glucosinolate, **3**; indole: indol-3-ylmethyl glucosinolate, **8**; aliphatic: 2-propenyl glucosinolate, **13**) to investigate the metabolism of these compounds (at least 3 replicates per developmental stage and glucosinolate, each pooled from 3 individuals). Pea is not a host of *P. cochleariae*, but this insect species feeds on its leaves when glucosinolates are applied^26^. Samples of whole insects, empty bodies (without guts), guts and faeces as well as leaves were analysed using an ultra-high performance liquid chromatograph coupled to a quadrupole time-of-flight mass spectrometer (UHPLC-QTOF-MS/MS), revealing different metabolites specifically found in samples of certain glucosinolate-feeding treatments (Tables 2, S2, S3). The glucosinolates that had been applied on the leaves were found in samples of both adults and larvae, while 2-phenylethyl glucosinolate, present in watercress, was not detectable in adults, but in larvae (Table S3; for larvae see^25^). Glucosinolates were mostly present in whole insects and faeces but also in gut samples of adults fed with treated pea leaves (Table S3). The glucosinolate identities were confirmed by comparison of retention times, ion types and mass spectra (MS and MS/MS mode) to reference standards, which showed characteristic fragments such as [SO_3_]^−^, [SO_4_]^−^ and [HSO_4_]^−^ ions and specific rearrangement products (Table S2)^27,28^.

**Table 2:** Glucosinolates and their corresponding putative breakdown metabolites detected by UHPLC-QTOF-MS/MS with molecular formulas and average retention times (RT) as well as the ion types, their observed mass-to-charge ratios (*m*/*z*) and ion formulas for the negative (ESI^−^) and positive (ESI^+^) electrospray ionisation mode, found in samples of *Phaedon cochleariae*.

ID	Metabolite	Molecular formula	RT [min] average	ESI^−^			ESI^+^		
				Ion type	Observed masses (*m*/*z*)	Ion formula	Ion type	Observed masses (*m*/*z*)	Ion formula
1	Benzyl glucosinolate	C_14_H_19_NO_9_S_2_	5.10	[M-H]^−^	408.0432	[C_14_H_18_NO_9_S_2_]^−^			
2	*N*-(Benzoyl) aspartic acid	C_11_H_11_NO_5_	5.88	[M-H]^−^	236.0567	[C_11_H_10_NO_5_]^−^	[M + H]^+^	238.0708	[C_11_H_12_NO_5_]^+^
3	4-Hydroxybenzyl glucosinolate	C_14_H_19_NO_10_S_2_	2.20	[M-H]^−^	424.0380	[C_14_H_18_NO_10_S_2_]^−^			
4	*N*-(4-Hydroxybenzoyl) aspartic acid	C_11_H_11_NO_6_	3.15	[M-H]^−^	252.0517	[C_11_H_10_NO_6_]^−^	[M + H]^+^	254.0661	[C_11_H_12_NO_6_]^+^
5	4-Hydroxybenzoic acid	C_7_H_6_O_3_	4.15	[M-H]^−^	137.0244	[C_7_H_5_O_3_]^−^	[M + H]^+^	139.0388	[C_7_H_7_O_3_]^+^
6	2-Phenylethyl glucosinolate	C_15_H_21_NO_9_S_2_	7.90	[M-H]^−^	422.0583	[C_15_H_20_NO_9_S_2_]^−^			
7	*N*-(Phenylacetyl) aspartic acid	C_12_H_13_NO_5_	6.75	[M-H]^−^	250.0724	[C_12_H_12_NO_5_]^−^	[M + H]^+^	252.0867	[C_12_H_14_NO_5_]^+^
8	Indol-3-ylmethyl glucosinolate	C_16_H_20_N_2_O_9_S_2_	6.50	[M-H]^−^	447.0546	[C_16_H_19_N_2_O_9_S_2_]^−^			
9	*N*-(1*H*-Indol-3-ylcarbonyl) glutamic acid	C_14_H_14_N_2_O_5_	8.45	[M-H]^−^	289.0828	[C_14_H_13_N_2_O_5_]^−^	[M + H]^+^	291.0974	[C_14_H_15_N_2_O_5_]^+^
10	*m*/*z* 463	C_16_H_20_N_2_O_10_S_2_	5.15	[M-H]^−^	463.0494	[C_16_H_19_N_2_O_10_S_2_]^−^			
11	*m*/*z* 190	C_10_H_9_NO_3_	7.25	[M-H]^−^	190.0509	[C_10_H_8_NO_3_]^−^			
12	Ascorbigen	C_15_H_15_NO_6_	8.10	[M-H]^−^	304.0828	[C_15_H_14_NO_6_]^−^	[M + H]^+^	306.0957	[C_15_H_16_NO_6_]^+^
13	2-Propenyl glucosinolate	C_10_H_17_NO_9_S_2_	1.45	[M-H]^−^	358.0274	[C_10_H_16_NO_9_S_2_]^−^			

Metabolite 6 shown in grey was only detected in larvae in a previous study ^25^. The glucosinolates were not found in ESI^+^ mode. Glucosinolates are given in bold, each followed by the corresponding putative breakdown metabolites. Further details are given in Supplement Table S2.

In samples of adults, the main metabolites of the benzenic glucosinolates (**1**, **3**, **6**) were identified as *N*-(benzoyl) aspartic acid (**2**, derived from **1**), *N*-(4-hydroxybenzoyl) aspartic acid (**4**, derived from **3**) and *N*-(phenylacetyl) aspartic acid (**7**, derived from **6**) (Tables 2, S2, Fig. 2), as has previously also been shown in larvae^25^. Common fragments of these aspartic acid conjugates in negative electrospray ionisation (ESI^−^) mode had a mass-to-charge ratio (*m*/*z*) of 115 ([C_4_H_3_O_4_]^−^, metabolites **2**, **4** and **7**) and an *m*/*z* of 132 ([C_4_H_6_NO_4_]^−^, metabolites **4** and **7**) (Table S2). When fed with 4-hydroxybenzyl glucosinolate-treated watercress, in addition to *N*-(4-hydroxybenzoyl) aspartic acid (**4**) 4-hydroxybenzoic acid (**5**) was detected in adult bodies and their faeces. The latter metabolite has previously also been found in larvae^25^. 4-Hydroxybenzaldehyde (**14**), previously found in larvae fed with 4-hydroxybenzyl glucosinolate-treated watercress leaves and in similar treated leaf samples^25^, was not detectable in samples of adults. All three metabolites (**4**, **5**, **14**) were not detectable in adults fed with 4-hydroxybenzyl glucosinolate-treated pea leaves (except for **4** in one sample of whole insects).

**Figure 1:**
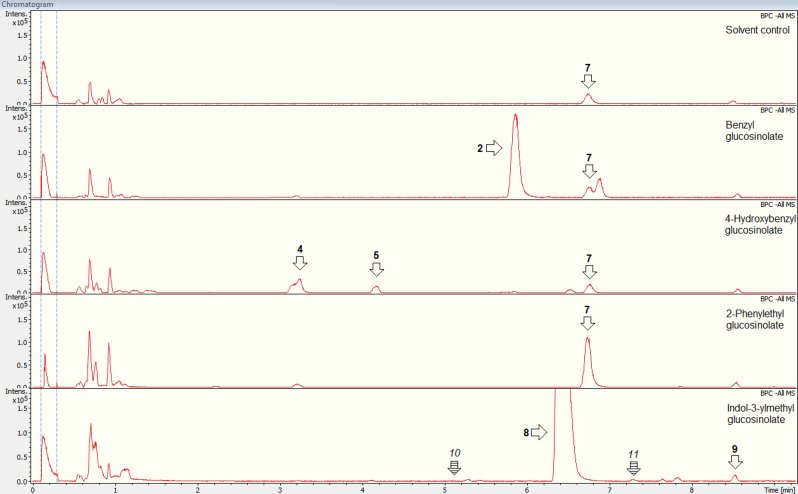
Representative chromatograms of extracts of faeces from adults of *Phaedon cochleariae*, fed with leaves of *Nasturtium officinale* or *Pisum sativum* treated with the solvent only, benzyl glucosinolate, 4-hydroxybenzyl glucosinolate, 2-phenylethyl glucosinolate (not directly fed, but major glucosinolate in watercress) or indol-3-ylmethyl glucosinolate, respectively. The glucosinolates and metabolites thereof are highlighted by arrows (or hatched arrows for the respective position in cases where no peaks can be seen); the numbers correspond to the metabolites listed in Tables 2 and S2.

In samples of some adults and in faeces of some larvae that were fed with leaves treated with indol-3-ylmethyl glucosinolate, the most abundant breakdown metabolite of this glucosinolate was putatively identified as *N*-(1*H*-indol-3-ylcarbonyl) glutamic acid (**9**), based on the fragments with an *m*/*z* of 116 ([C_8_H_6_N]^−^), an *m*/*z* of 128 ([C_5_H_6_NO_3_]^−^) and an *m*/*z* of 146 ([C_5_H_8_NO_4_]^−^) in ESI^−^ mode (Table S2, Fig. 1). Two further metabolites [ions with an *m*/*z* of 463 (**10**) and an *m*/*z* of 190 (**11**), respectively] were primarily detected in samples of adults fed with pea treated with indol-3-ylmethyl glucosinolate (**11**, also in one faecal sample of adults fed with watercress), but could not be identified. Moreover, ascorbigen (**12**) was found in most adult and few larval samples of individuals that were fed with leaves treated with the indole glucosinolate (Table S3) as well as in all the corresponding treated leaves of watercress, but only in one of the pea leaf samples. Ascorbigen was identified by comparison with a reference standard, showing characteristic fragments with an *m*/*z* of 115 ([C_4_H_3_O_4_]^−^), an *m*/*z* of 116 ([C_8_H_6_N]^−^) and an *m*/*z* of 244 ([C_13_H_10_NO_4_]^−^; Tables 2, S2).

The metabolism of the aliphatic glucosinolate (**13**) by *P. cochleariae* could not be elucidated by UHPLC-QTOF-MS/MS, because no metabolites were detected exclusively in those adults and larvae that were fed with 2-propenyl glucosinolate-treated leaves. As alternative approach, volatiles were collected on polydimethylsiloxane (PDMS) tubes while individuals were fed with 2-propenyl glucosinolate-treated or solvent-treated watercress leaves and from leaves only. Subsequent thermal desorption-gas chromatography-mass spectrometry (TD–GC–MS) of these tubes revealed the release of 2-propenyl isothiocyanate when insects were fed with glucosinolate-treated leaves as well as, to a lesser extent (about 18–33%), from those leaves themselves. In addition, hydrolysis products of the main glucosinolate in watercress, 2-phenylethyl glucosinolate, were released when insects were fed with either the glucosinolate- or the solvent-treated leaves and from the leaves only, with 20- (leaves) up to 83-fold (adults with leaves) higher concentrations of 2-phenylethyl isothiocyanate compared to 3-phenylpropanenitrile. 2-Propenyl isothiocyanate, 2-phenylethyl isothiocyanate and 3-phenylpropanenitrile were identified by comparison of Kováts retention indices and mass spectra to authentic standards and the Pherobase database (Table 3). The nitrile formed from 2-propenyl glucosinolate, 3-butenenitrile, was also available as standard but was not detectable with the used method.

**Table 3:** Volatiles trapped on PDMS tubes and measured by TD–GC–MS with molecular formulas, monoisotopic masses, observed mass-to-charge ratios (*m*/*z*) of molecular ions and fragments, retention times (RT), Kováts retention indices (KI; GC–MS data of the current study and entries in the Pherobase database, respectively), occurrence (out of 3 replicates) and peak areas (mean ± standard deviation) in samples of adults and larvae of *Phaedon cochleariae* fed with 2-propenyl glucosinolate-treated or solvent-treated watercress leaves (containing 2-phenylethyl glucosinolate) as well as of the corresponding leaves.

	2-Propenyl glucosinolate		2-Phenylethyl glucosinolate	
	2-Propenyl isothiocyanate	3-Butenenitrile	2-Phenylethyl isothiocyanate	3-Phenylpropanenitrile
*Characteristics*				
Molecular formula	C_4_H_5_NS	C_4_H_5_N	C_9_H_9_NS	C_9_H_9_N
Monoisotopic mass (Da)	99.01	67.04	163.05	131.07
Observed (*m*/*z*)	39, 41, 99	–	91, 105, 163	91, 131
RT [min]	7.09	–	24.81	18.67
KI current study	883	–	1469	1242
KI Pherobase (column)	887 (HP-5MS)	–	1465 (HP-5MS)	1246 (BPX-5)
*Adults*				
2-Propenyl glucosinolate	3/3	–	3/3	2/3
Peak area	4.82 ± 1.90	–	9.16 ± 2.59	0.11 ± 0.02
Occurrence	0/3	–	3/3	3/3
Peak area	0	–	5.07 ± 1.46	0.08 ± 0.03
*Larvae*				
2-Propenyl glucosinolate	3/3	–	3/3	3/3
Peak area	8.73 ± 7.02	–	10.12 ± 7.97	0.24 ± 0.04
Occurrence	0/3	–	3/3	3/3
Peak area	0	–	5.24 ± 1.71	0.12 ± 0.01
*Leaves*				
2-Propenyl glucosinolate	3/3	–	3/3	3/3
Peak area	1.61 ± 1.36	–	1.83 ± 1.03	0.09 ± 0.03
Occurrence	0/3	–	3/3	3/3
Peak area	0	–	2.71 ± 1.47	0.09 ± 0.01

All metabolites were identified by comparison to reference standards (3-butenenitrile was not detectable).

### Metabolism of glucosinolate hydrolysis products in *P. cochleariae*

A further set of feeding experiments was performed applying equal amounts of available isothiocyanates or nitriles on leaves of watercress and offering them to adults and larvae. Whole insects and faeces were analysed by UHPLC-QTOF-MS/MS to investigate whether the main breakdown metabolites derived from the glucosinolates as described above are also formed after feeding any of these hydrolysis products. The isothiocyanates or nitriles themselves were not detectable with this analytical platform. In whole insects and faeces of both adults and larvae fed with leaf discs treated with either benzyl isothiocyanate or phenylacetonitrile (derived from benzyl glucosinolate) the main metabolite *N*-(benzoyl) aspartic acid was detectable (Table 4). Likewise, *N*-(phenylacetyl) aspartic acid occurred in samples of insects when fed with leaves treated with 2-phenylethyl isothiocyanate or 3-phenylpropanenitrile (derived from 2-phenylethyl glucosinolate). The amino acid conjugates occurred in about two to twelve times higher concentrations in samples of insects fed with leaves treated with isothiocyanates than in those fed with leaves with the corresponding nitriles. An exception were larvae fed with leaves treated with hydrolysis products of 2-phenylethyl glucosinolate, in which the amino acid conjugate was about 1.3- and 2.1-fold higher concentrated in whole insects and faeces, respectively, when individuals were fed with 3-phenylpropanenitrile- compared to 2-phenylethyl isothiocyanate-treated leaves.

**Table 4:** Peak heights (multiplied with 1000, detected with UHPLC-QTOF-MS/MS) and occurrence of main (putative) metabolites found in adults and larvae of *Phaedon cochleariae* (whole insects or faeces; 3–5 replicates) after feeding on watercress leaves treated with isothiocyanates or nitriles (treatment) that are hydrolysis products of the indicated glucosinolates and consumed leaf areas.

Glucosinolate	Treatment	Main (putative) metabolites	Adults					Larvae				
			Mean peak height (± SD)		Occurrence		Mean consumed leaf area [%] (± SD)	Mean peak height (± SD)		Occurrence		Mean consumed leaf area [%] (± SD)
			Whole	Faeces	Whole	Faeces		Whole	Faeces	Whole	Faeces	
Benzyl glucosinolate	Benzyl isothiocyanate	*N*-(benzoyl) aspartic acid	9.6 ± 8.1	6.5 ± 2.3	5/5	5/5	9.7 ± 4.8	2.4 ± 0.4	3.7 ± 1.3	3/3	3/3	30.8 ± 14.5
	Phenylacetonitrile	*N*-(benzoyl) aspartic acid	2.8 ± 2.1	1.9 ± 0.7	3/3	3/3	13.8 ± 4.8	1.4 ± 0.7	0.8 ± 0.3	3/3	3/3	32.7 ± 7.3
4-Hydroxybenzyl glucosinolate	4-hydroxyphenylacetonitrile	*N*-(4-hydroxybenzoyl) aspartic acid	0.1 ± 0.2	0.2 ± 0.3	1/3	1/3	22.2 ± 6.3	1.7 ± 1.0	3.0 ± 2.2	3/3	3/3	34.2 ± 6.8
2-Phenylethyl glucosinolate	2-Phenylethyl isothiocyanate	*N*-(phenylacetyl) aspartic acid	56.1 ± 76.8	26.1 ± 26.1	5/5	5/5	17.4 ± 15.1	6.1 ± 0.5	6.0 ± 3.6	3/3	3/3	33.4 ± 7.5
	3-Phenylpropanenitrile	*N*-(phenylacetyl) aspartic acid	4.6 ± 1.3	2.6 ± 0.8	3/3	3/3	14.1 ± 4.0	7.9 ± 1.5	12.7 ± 3.6	3/3	3/3	48.5 ± 9.5
Indol-3-ylmethyl glucosinolate	Indole-3-acetonitrile	*N*-(1*H*-indol-3-ylcarbonyl) glutamic acid	0 ± 0	0.1 ± 0.1	0/3	1/3	35.1 ± 0.8	0 ± 0	0 ± 0	0/3	0/3	34.1 ± 5.3

Peak heights and consumed leaf areas are given as means ± standard deviations.

For 4-hydroxybenzyl glucosinolate and indol-3-ylmethyl glucosinolate only the corresponding nitriles (4-hydroxyphenylacetonitrile and indole-3-acetonitrile) were available. When fed with 4-hydroxyphenylacetonitrile-treated leaves, the main breakdown metabolite *N*-(4-hydroxybenzoyl) aspartic acid was found in only one third of the adult samples in low intensities, but was detectable in all larval samples (Table 4). In comparison, *N*-(1*H*-indol-3-ylcarbonyl) glutamic acid was only detectable in the faeces of one adult replicate, but not in larvae when fed with indole-3-acetonitrile-treated leaves.

### Myrosinase activities in adult body parts

The amino acid conjugates (**2**, **4**, **7**, and **9**) derived from the glucosinolates were not only found in individuals fed with watercress-treated leaves, but also in those fed with pea-treated leaves (Table S3), although pea lacks myrosinases^25^. To test whether adult leaf beetles have their own myrosinase activity, the guts and the remaining bodies of freshly hatched adult beetles (starved for 24 h) were separately examined in a spectrophotometric assay, but no myrosinase activity could be detected. Likewise, no activity had been found in freshly moulted larvae^25^. After adults were fed with watercress leaves, myrosinase activity towards 2-propenyl glucosinolate as substrate could be detected in their guts [0.012 ± 0.014 nmol glucose (µg protein * min)^−1^, mean ± SD, n = 3]. In the remaining bodies of adults fed with watercress no myrosinase activity was detectable towards the substrates benzyl glucosinolate, 4-hydrobenzyl glucosinolate and 2-propenyl glucosinolate. Adults fed with pea leaves did not show any myrosinase activity in line with the fact that we could also not measure any myrosinase activity in pea leaves in our previous study^25^.

## Discussion

**Figure 2:**
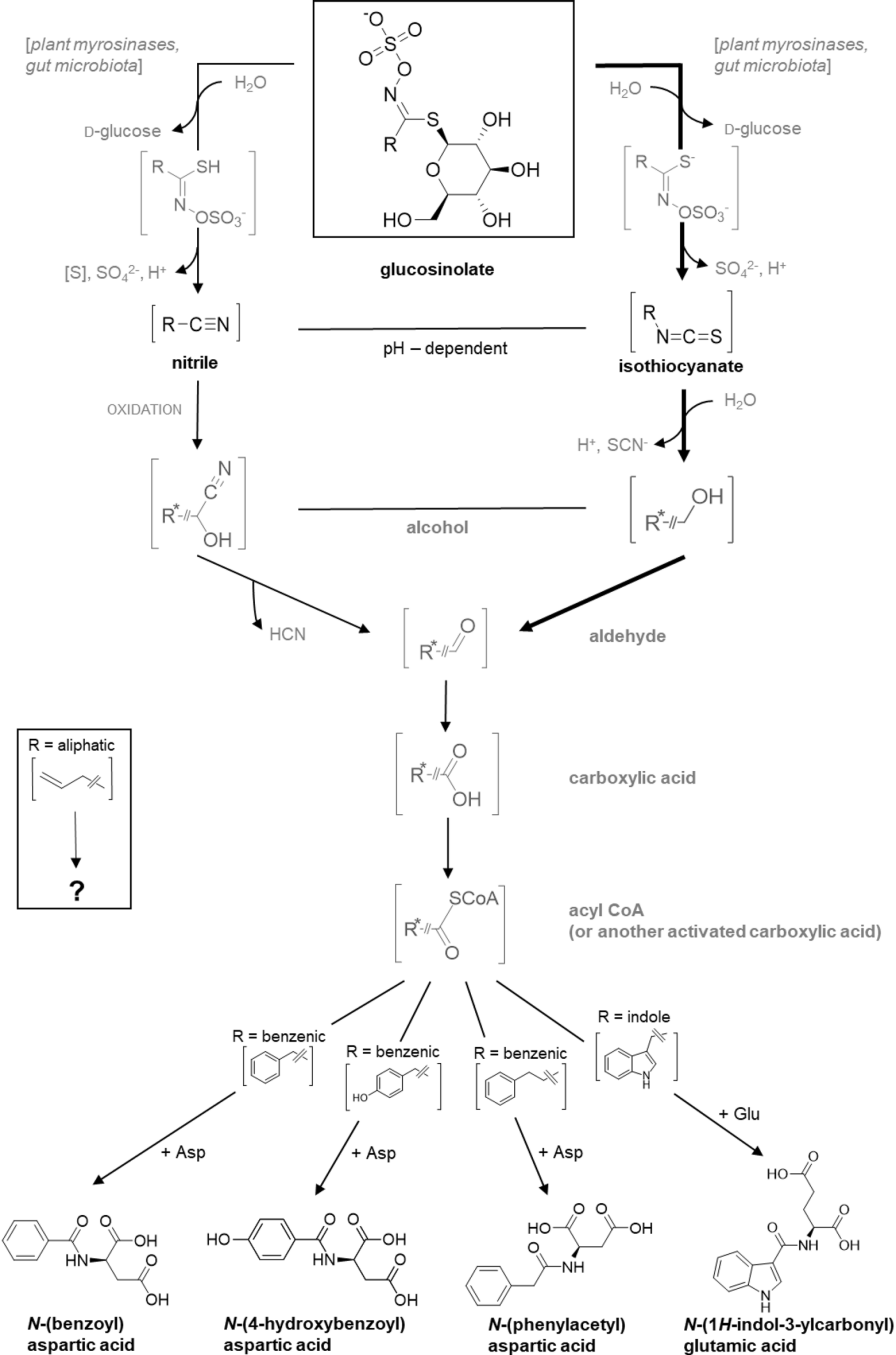
Suggested metabolism of structurally different glucosinolates (benzenic, indole and aliphatic) in *Phaedon cochleariae*. The reactions are based on metabolites found in adults and larvae fed with watercress or pea leaves treated with glucosinolates or their hydrolysis products (isothiocyanates or nitriles). In the first step, glucosinolates are hydrolysed to isothiocyanates and nitriles, with isothiocyanate formation being predominant (thicker black lines). Subsequently, an alcohol is oxidised to an aldehyde, which further reacts to a carboxylic acid. In the next step, the carboxylic acid is metabolised to an acyl coenzyme A (CoA)-complex (or another activated carboxylic acid) and further conjugated with the amino acids aspartic acid or glutamic acid, dependent on the side chain (benzenic or indole). The metabolism of the aliphatic glucosinolate is still unclear and therefore indicated in a separate box with the reaction ending in a question mark. Putative intermediates are shown in grey. If the molecular residue is marked with an asterisk (R*), it is defined as residue minus one methylene group.

**Figure 3:**
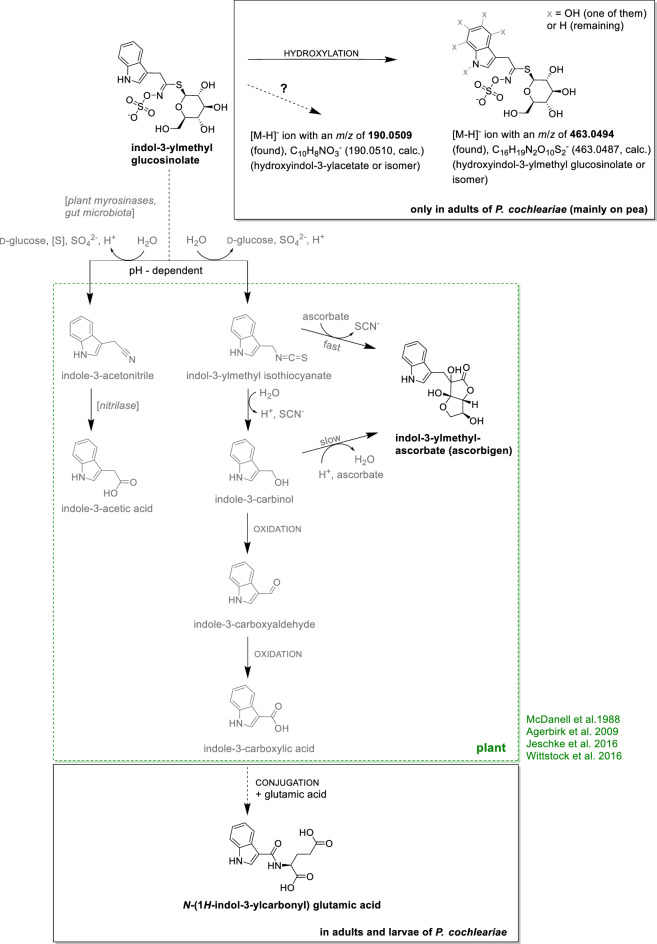
Suggested indole glucosinolate metabolism in *Phaedon cochleariae*. Experiments with adults and larvae fed with indole glucosinolate-treated watercress and pea leaves provided metabolites (black) indicating a possible course of reactions. In the first step, hydrolysis reactions by plant myrosinases or gut microbiota probably result in the formation of indol-3-ylmethyl isothiocyanate and indole-3-acetonitrile. The following steps are known at least in plants where either the nitrile is metabolised by a nitrilase to indole-3-acetic acid or the isothiocyanate is hydrolysed to indole-3-carbinol, which is further oxidised to indole-3-carboxyaldehyde and then to indole-3-carboxylic acid. In the next step, indole-3-carboxylic acid or an activated form of it is probably conjugated with glutamic acid resulting in *N*-(1*H*-indol-3-ylcarbonyl) glutamic acid. In addition, the isothiocyanate and the carbinol are further metabolised with ascorbate to indol-3-ylmethylascorbate (ascorbigen). The reaction steps described above are based on literature and have been mainly found in plants, but nitrile formation was partly also found in insects^13,14,31,58^. At least conjugations with amino acids are also known to occur in insects (Tables 1, S1). A second potential metabolism of indol-3-ylmethyl glucosinolate, observed only in adults in the current study, appears to be a hydroxylation reaction resulting in an ion with an *m*/*z* of 463 (probably hydroxyindol-3-ylmethyl glucosinolate or isomer); in addition, an ion with an *m*/*z* of 190 (probably a hydroxyindol-3-yl acetate or isomer) was found. Putative intermediates are shown in grey.

Our previous study with *P. cochleariae*^25^ revealed that larvae of this leaf beetle species use a detoxification metabolism for benzenic glucosinolates that has not been described for other insect species yet (Tables 1, S1), namely a conjugation of an intermediate probably derived from isothiocyanates and/or nitriles with aspartic acid. In the present study, we tested whether individuals metabolise structurally different glucosinolates with distinct properties based on their side chains in different ways and whether the metabolism differs between adults and larvae. Comparative metabolomics of samples of adults and larvae which were fed with different glucosinolates supported our first hypothesis: aspartic acid conjugates of putative glucosinolate-derived intermediates (**2**, **4**, **7**; Tables 2, S2) were found after individuals were fed with different benzenic glucosinolates (**1**, **3**, **6**). In contrast, the major metabolite found after individuals were fed with an indole glucosinolate (**8**) was a glutamic acid conjugate, *N*-(1*H*-indol-3-ylcarbonyl) glutamic acid (**9**, Fig. 2). Contrary to our second hypothesis, the glucosinolate metabolism did not differ much between adults and larvae of *P. cochleariae*. However, when fed particularly with pea leaves treated with indol-3-ylmethyl glucosinolate, two additional features with an *m*/*z* of 463 (**10**) and an *m*/*z* of 190 (**11**), were detected only in adults but not in larvae. The ion with an *m*/*z* of 463 may belong to an indol-3-ylmethyl glucosinolate with an additional hydroxyl-group somewhere at the indole ring or at the methylene group. For such a metabolite, an [M-H]^−^ ion of *m*/*z* 463.0476 is expected, while the measured ion had an *m*/*z* of 463.0494 (Table S2). The unidentified *m*/*z* 190 ion in the same samples could similarly correspond to a hydroxyindol-3-yl acetate (or isomer), meaning that both unidentified metabolites may be due to a single metabolic step, namely a hydroxylation of indol-3-ylmethyl groups at an undetermined position. A direct hydroxylation of the glucosinolate may thus take place in adults in addition to the amino acid conjugation of a glucosinolate-derived intermediate (Fig. 3). A conversion of one glucosinolate to another has previously been reported for adults of the leaf beetle *Phyllotreta armoraciae*, which convert the aliphatic 4-(methylsulfinyl)butyl glucosinolate to 4-(methylthio)butyl glucosinolate^29^.

Furthermore, ascorbigen (**12**) was detected in adults and larvae of *P. cochleariae* when fed with indol-3-ylmethyl glucosinolate-treated watercress or pea leaves as well as in the treated watercress leaves, but just in one pea leaf sample in low concentrations (Tables 2, S3, Fig. 3). Ascorbate is involved in the iron uptake and transport in plants and can donate electrons, thereby acting as scavenger of free radicals^30^. When the tissues of Brassicaceae plants containing indol-3-ylmethyl glucosinolate are disrupted, the plant myrosinases hydrolyse this glucosinolate to the corresponding unstable isothiocyanate, which may further metabolise to indole-3-carbinol. Both indole-3-methyl isothiocyanate and indole-3-carbinol can be conjugated with ascorbate to ascorbigen^11,31^. In insects, ascorbate has several functions, including roles in the energy balance, immune responses as well as protection against oxidant plant compounds^32^. Individuals of *P. cochleariae* may use ascorbate from their food plant for similar purposes and, similar to the metabolic steps known from plants (see above), they may conjugate indol-3-ylmethyl isothiocyanate and/or indole-3-carbinol with ascorbate. Ascorbigen can act deterrent, for example, against the aphid *Myzus persicae* (Hemiptera: Aphididae)^33^, but does not seem to act deterrent against *P. cochleariae*, since both adults and larvae did not feed less on the leaves treated with indole glucosinolate than on leaves of the other treatments (Table S4).

The metabolism of the aliphatic 2-propenyl glucosinolate by *P. cochleariae* is still unclear, because we were not able to identify a potential breakdown metabolite in adults or larvae. This may be due to the instability of isothiocyanates and nitriles under certain conditions^34–36^. Furthermore, intermediates such as carboxylic acid, found for other glucosinolates, may not be formed from this aliphatic glucosinolate due to specific properties of enzymes along the pathway. In a few other generalist and specialist insect species it has been shown that 2-propenyl glucosinolate is either sequestered, desulfated or that the corresponding isothiocyanate is conjugated with glutathione (Tables 1, S1).

The question arises why *P. cochleariae* individuals specifically use aspartic acid and glutamic acid for the conjugation of intermediates derived from benzenic and indole glucosinolates, respectively. Asparagine, aspartic acid, glutamine and glutamic acid belong to the non-essential amino acids in insect diets^37^ and may thus be preferentially used for conjugation reactions. Individuals of *P. cochleariae* may rather take up these amino acids from their host plants, because their synthesis from metabolites lacking nitrogen may be metabolically more costly. Indeed, watercress and pea both contain relatively high concentrations of aspartic acid and glutamic acid compared to other amino acids^38–40^. Furthermore, in the insects aspartic acid and glutamic acid may be, if required, produced from the amides asparagine and glutamine, respectively. These amides are probably available in sufficient concentrations for conversion to acidic amino acids, because they are the major transport forms of amino acids in different plant parts of several plant species^41,42^ and can be induced if plants are stressed, for example, by abiotic factors or antagonists (pathogens, herbivores)^37,42,43^.

The metabolites produced after feeding structurally different glucosinolates were not only detected in individuals fed with watercress leaves, but also in those fed with glucosinolate-treated pea leaves (Table S3). Our previous study of *P. cochleariae* showed that freshly-moulted larvae as well as pupae do not show any own myrosinase activity^25^. Likewise, no myrosinase activity could be revealed in adults in the present study. In the chrysomelid *Psylliodes chrysocephala*, gut microbiota seem to be involved in isothiocyanate detoxification, because when they were suppressed, adults showed about 11-fold higher levels of isothiocyanates than the control group^44^. Likewise, the gut microbiome in humans and other vertebrates contributes to the production of isothiocyanates and nitriles in the absence of plant myrosinase activity^45–48^. In *P. cochleariae,* next to microbiota also a gregarine species occurs in the gut^49^. Gregarines can cover the whole range of symbiotic relationships from parasitism to mutualism, based on the environmental and host conditions^50^. It remains to be studied whether gregarines are involved in the detoxification of plant metabolites taken up by *P. cochleariae*.

To elucidate whether the glucosinolate metabolism in *P. cochleariae* happens via isothiocyanates and/or nitriles, adults and larvae were fed with isothiocyanate- or nitrile-treated watercress leaves and analysed for the presence of the breakdown metabolites of the corresponding glucosinolates. Preliminary tests with different concentrations of the hydrolysis products showed that *P. cochleariae* fed on treated leaves even when higher concentrations were used, indicating that these compounds do not act repellent or deterrent for the beetles. Rather, 2-phenylethyl isothiocyanate seems to act as an olfactory cue for host plant location^51^. The analyses of the insect samples in the present study revealed that the detoxification reactions can take place via both isothiocyanates and nitriles, because the corresponding amino acid conjugates could be found in adults and larvae fed with both types of hydrolysis products of benzyl glucosinolate and 2-phenylethyl glucosinolate (Table 4). However, the concentrations of the amino acid conjugates were two to twelve times higher in most individuals (especially in adults) fed with isothiocyanate- compared to nitrile-treated leaves, while they consumed comparable leaf amounts (Table 4). In contrast, larvae fed with hydrolysis products of 2-phenylethyl glucosinolate showed higher concentrations of the amino acid conjugate when fed with the corresponding nitrile. This indicates that there may be slight differences in the metabolism of glucosinolate hydrolysis products by adults and larvae. For 4-hydroxybenzyl glucosinolate and indol-3-ylmethyl glucosinolate only the nitriles were available and feeding of them resulted in very low concentrations of the corresponding amino acid conjugates. Thus, the reactions may primarily take place via isothiocyanates. The fact that ascorbigen was detectable in feeding experiments with indol-3-ylmethyl glucosinolate may also support the assumption, since ascorbigen is formed by conjugation of the corresponding isothiocyanate (indol-3-ylmethyl isothiocyanate) with ascorbate^11^. In other specialised Chrysomelidae species, such as *Phyllotreta striolata* and *Psylliodes chrysocephala*, but also in many other feeding generalist and specialist species, the metabolism likewise mainly occurs via isothiocyanates (^18,19,44^, Tables 1, S1).

While individuals were feeding on 2-propenyl glucosinolate-treated watercress leaves that contained 2-phenylethyl glucosinolate, higher concentrations of 2-propenyl isothiocyanate and 2-phenylethyl isothiocyanate than of 3-phenylpropanenitrile were detectable. The corresponding nitrile of 2-propenyl glucosinolate, 3-butenenitrile, was not detectable with TD–GC–MS. Moreover, both isothiocyanates were found in higher concentrations when insects were feeding on the leaves than in volatile samples collected from treated leaves only. Higher concentrations of isothiocyanates released from insect-plant complexes than from undamaged leaves may either be due to the metabolism of *P. cochleariae* or the plant tissue disruption while feeding, bringing the glucosinolates into contact with plant myrosinases. In the leaf beetle *P. armoraciae* higher concentrations of 2-propenyl isothiocyanate were found in the headspace of beetles fed with wild-type plants of *Arabidopsis thaliana* compared to beetles fed with mutants, in which plant myrosinase activity was suppressed (^52^, Tables 1, S1). However, 2-propenyl isothiocyanate was also found in the latter, thus isothiocyanate production can occur independently of plant myrosinases in *P. armoraciae*, which may likewise be the case in *P. cochleariae*.

In summary, *P. cochleariae* has evolved a novel way to metabolise hydrolysis products of structurally different glucosinolates. Both adults and larvae use aspartic acid to conjugate intermediates formed from the hydrolysis products of benzenic glucosinolates and glutamic acid for indole glucosinolate metabolism, independently of plant myrosinase activity. Whether gut microbiota or gregarines are involved in the metabolism needs to be investigated in future studies.

## Materials and methods

### Insect and plant maintenance

Individuals of *P. cochleariae* were taken from our laboratory rearing (for details see^25^) and kept in ventilated plastic boxes (20 × 20 × 6.5 cm; 50–100 individuals per box) in a climate cabinet at 20 °C, 65% r.h. and 16:8 h light:dark. Insects were fed with shoots of watercress. Plants of watercress (seeds from Volmary GmbH, Münster, Germany) and pea (seeds from Kiepenkerl, Bruno Nebelung GmbH, Konken, Germany) used for the rearing and/or for feeding experiments (see below) were 6–8 weeks old, not flowering and grown in a greenhouse (60% r.h., 16:8 h light:dark). The glucosinolate profile of these watercress plants was dominated by 2-phenylethyl glucosinolate (~ 89%), followed by 7-(methylsulfinyl)heptyl glucosinolate (~ 6%) and indol-3-ylmethyl glucosinolate as well as 8-(methylsulfinyl)octyl glucosinolate occurring in low amounts (~ 1% or less). The use of the plants in the present study complies with international, national and institutional guidelines.

### Feeding bioassays with glucosinolates and hydrolysis products

The metabolism of structurally different glucosinolates was investigated by providing individuals of *P. cochleariae* with leaf discs (2 cm diameter) of either watercress or pea. Leaf discs were treated with solutions (50 µL, 40 mM) of either benzyl glucosinolate, 4-hydroxybenzyl glucosinolate, indol-3-ylmethyl glucosinolate or 2-propenyl glucosinolate (all > 95–99%, Phytoplan Diehm & Neuberger GmbH, Heidelberg, Germany), dissolved each in a mixture of methanol (MeOH), millipore water (H_2_O) and dichloromethane (DCM) (64:30:6, v:v:v; organic solvents from Fisher Scientific, Loughborough, UK), or treated with the solvent only (as in^25^). Discs were kept for 40 min on moistened filter paper under a fume hood for solvent evaporation and then offered in Petri dishes (55 mm diameter) to *P. cochleariae*.

All mentioned glucosinolates were offered to adults (24 h after emergence), while experiments with larvae (5 h after moult to second instar) were performed with the two glucosinolates indol-3-ylmethyl glucosinolate and 2-propenyl glucosinolate only, because the larval metabolism of benzenic glucosinolates has already been investigated^25^. Individuals had been starved since their last moults. The treated leaf discs were offered to groups of three individuals for 24 h (watercress) or 48–72 h (pea) (6 replicates per plant species and test solution) to seek an uptake of at least 10%. Afterwards, from half of the groups individuals were dissected on ice into guts (“gut”) and remaining body tissues (“body”). The remaining individuals were transferred into 2 mL Eppendorf tubes without food for 3 h to collect their faeces (“faeces”), and individuals with emptied guts were collected separately (“whole”). Thus, per treatment combination, three replicates pooled from three individuals were collected. In addition, leaf discs treated with the same glucosinolates or solvent but without insects were kept in Petri dishes for the same experimental period (n = 3 per treatment group and plant species).

To determine which glucosinolate hydrolysis products mainly lead to the final main breakdown metabolites found in *P. cochleariae*, individuals were fed with available isothiocyanates or nitriles. Therefore, groups of three individuals (3 replicates per treatment) were starved for 3 h and afterwards fed for 5 h with leaf discs of watercress, on which solutions (50 µL, 40 mM) of either benzyl isothiocyanate, phenylacetonitrile (benzyl cyanide) (98%, Sigma Aldrich Chemie GmbH, Steinheim, Germany), 4-hydroxyphenylacetonitrile, indole-3-acetonitrile (98%, Acros Organics, New Jersey, USA), 2-phenylethyl isothiocyanate, 3-phenylpropanenitrile (98%, Alfa Aesar, Kandel, Germany), dissolved in MeOH, H_2_O and DCM (64:30:6, v:v:v) or the solvent only were applied. The feeding duration was restricted to 5 h, as isothiocyanates and nitriles gradually evaporate over time. After the feeding period, samples of whole individuals and faeces were collected as described above. All insect samples were frozen in liquid nitrogen, stored at −80 °C and lyophilised. The freeze-dried samples were than extracted and measured with UHPLC-QTOF-MS/MS (see below).

Metabolites derived from 2-propenyl glucosinolate were not detectable in the insect samples by UHPLC-QTOF-MS/MS analyses. Thus, to test for the release of the corresponding isothiocyanate and/or nitrile, volatiles were collected while individuals were feeding on leaves. Adults and 2nd instar larvae that had fed for some time on plants were starved for 3 h and afterwards fed with 2-propenyl glucosinolate- or solvent-treated watercress leaf discs (n = 3, pool of 3 individuals) in glass dishes (9.5 cm diameter) for 4 h. In separate dishes, leaf discs with 2-propenyl glucosinolate or solvent were sampled for 4 h without insects. Volatiles released by the insects and leaves were trapped on tubes of absorbent PDMS (length 5 mm; diameter: internal 1 mm, external 1.8 mm; Carl Roth, Karlsruhe, Germany) that had been cleaned beforehand in an acetonitrile:MeOH solution (4:1, v:v) for 1 d and then heated up to 230 °C for 30 min using a conditioning program with a 60 mL min^−1^ helium flow^53,54^. Three tubes per dish were placed in a gauze mesh bag to prevent direct contact with the insects or leaves. In separate glass dishes, volatiles were collected from either blanks (only PDMS tubes) or 2-phenylethyl isothiocyanate, 3-phenylpropanenitrile, 3-butenenitrile (allyl cyanide) (all three 98%, Alfa Aesar) or 2-propenyl isothiocyanate (allyl isothiocyanate) (98%, Sigma Aldrich) (1 µL, standard:solvent mixture, 1:200, v:v) applied on the surfaces of the glass dishes. Additionally, a filter paper (1 cm^2^) with 1-bromodecane (2 µL, 20 ng µL^−1^; 98%, Sigma Aldrich) was placed in each dish, which was used as internal standard for normalisation of the peak areas. PDMS tubes were stored at -80 °C and measured using TD–GC–MS (see below)*.*

### Identification of metabolites in insect and plant samples via UHPLC-QTOF-MS/MS

For the identification of glucosinolates and their breakdown metabolites in insect and plant material, samples were extracted in 90% MeOH and analysed using UHPLC-QTOF-MS/MS as described in Friedrichs et al.^25^ (for details of extraction, instrument parameters and data analyses see Supplement S5A).

To screen for potential breakdown metabolites of the glucosinolates, the intensities of molecular features (normalised using the internal standard hydrocortisone) in the different treatment groups (plant species, treatment of the leaf disc, type of insect sample) were compared. In the insect datasets only features were retained: (1) whose mean intensity (peak height) in at least one treatment group was at least 50 times greater than the mean intensity in the blank samples, (2) which were present in at least two of the three replicates in at least one treatment group, (3) whose fold changes were > 2 or features only occurred in samples from individuals fed with the glucosinolate (not in the control individuals), (4) whose mean intensity was > 1000 counts in at least one glucosinolate treatment group.

To identify the metabolites **1**–**13** (Tables 2, S2), the samples with the highest feature intensities were measured again by UHPLC-QTOF-MS/MS with multiple reaction monitoring (MRM) in negative and/or (with capillary voltage 4500 V) positive ESI mode and/or at a lower spectral rate (2 Hz). Specific collision energies and isolation widths for the parent ion *m*/*z* were used to obtain MS/MS spectra. The SmartFormula (manually and 3D) function in DataAnalysis was applied to create molecular formulas of parent and daughter ions using their accurate *m*/*z* values and isotopic patterns. MetFrag^55^ was used for *in-silico* fragmentation (compound lists from: PubChem) to obtain potential structural formulas, while spectra were additionally compared with entries in the MassBank of North America (https://mona.fiehnlab.ucdavis.edu/). Furthermore, retention times, UV/VIS, MS and MS/MS spectra of metabolites were compared to those of reference standards of an in-house database.

### Volatile collection and TD–GC–MS measurements of isothiocyanates and nitriles

PDMS tubes were analysed by TD–GC–MS (TD 30–GC 2010 Plus – MS QP2020, Shimadzu, Kyoto, Japan) in electron impact ionisation mode. The GC was equipped with a VF-5 MS column (30 m × 0.25 mm ID, 10 m guard column, Varian, Agilent Technologies, Santa Clara, Kalifornien, USA) operated with helium as carrier gas. Desorption of volatiles on PDMS tubes was done at 210 °C under a flow rate of 60 mL min^−1^ and adsorption took place at −20 °C for 8 min in a Tenax® cryo-trap. Compounds were re-desorbed for 8 min at 250 °C, transferred to the GC in a 1:1 split mode with the same temperature setting and a column flow rate of 1.6 mL min^−1^. The GC oven temperature was set to 50 °C for 5 min, increased to 150 °C with 5 °C min^−1^ and then to 280 °C with 10 °C min^−1^ (total duration 38 min). Line spectra (*m*/*z* of 30–400) were obtained in quadrupole MS mode at 70 eV. An alkane standard mixture (C8–C20, Sigma Aldrich) was used to calculate Kováts retention indices (KI)^56^. Identification of compounds was performed by comparing the KI and mass spectra to authentic standards, the National Institute of Standards and Technology NIST 2014 database and the Pherobase website^57^. Compounds were quantified using *m*/*z* values of characteristic fragments.

### Myrosinase activity measurements

To measure myrosinase activities in insect samples, freshly hatched adults were kept for 24 h without food, then either sampled directly or fed with untreated leaf discs of watercress or pea for 24 h. Afterwards, adults were dissected on ice into guts and remaining bodies and frozen at -20 °C. Two to six individuals per treatment were measured for their myrosinase activities as described in^25^ (for details see Supplement S5B).

## Supplementary Information

Supplementary Information 1.

Supplementary Information 2.

Supplementary Information 3.

Supplementary Information 4.

Supplementary Information 5.

## Data Availability

All data generated or analysed during this study are included in this published article [and its supplementary information files]. The metabolic data will be available in MetaboLights (https://www.ebi.ac.uk/metabolights/, accession number MTBLS3565).
