# Supplementary material for: Unique metabolism of different glucosinolates in larvae and adults of a leaf beetle specialised on Brassicaceae
Source: Sci Rep. 2022 Jun 28;12:10905. doi: 10.1038/s41598-022-14636-6 (PMC9240079; doi:10.1038/s41598-022-14636-6)
Supplement: Supplementary file 5 — Supplementary Information 5. [file 41598_2022_14636_MOESM5_ESM.pdf]

Supplement 5A: ***Detailed methods for measurement and identification of metabolites in insect and plant samples via UHPLC-QTOF-MS/MS.***

Samples were extracted on ice in ice-cold MeOH (300 µL, 90%, v:v) containing hydrocortisone (65,1 µg in 300 µL, >98%, Sigma-Aldrich Chemie GmbH, Steinheim, Germany) as internal standard. The extracts were vortexed for 1 min and centrifuged for 5 min at 4 °C. The supernatants were filtered with 0.2 µm polytetrafluoroethylene membrane syringe filters (Phenomenex, Torrance, CA, USA) and stored at -80 °C. Samples were analysed with an UHPLC-QTOF-MS/MS system (UHPLC: Dionex UltiMate 3000, Thermo Fisher Scientific, San José, CA, USA; Q-TOF: compact, Bruker Daltonics, Bremen, Germany) connected with a Kinetex XB-C18 column (1.7 µm, 150 x 2.1 mm, with guard column, Phenomenex), using a gradient of water (eluent A) to acetonitrile (eluent B; LC-MS grade, 99.95%, Th. Geyer GmbH & Co. KG, Renningen, Germany), both acidified with formic acid (FA, 0.1 %, v:v; eluent additive for LC-MS, ~98%, Sigma-Aldrich). The gradient went from 2% to 30% B within 20 min and then increased to 75% B within 9 min with a flow rate of 0.5 mL min^{-1} at 45 °C, followed by column cleaning and equilibration.

The electrospray ionisation (ESI) source was operated in negative mode. MS and MS/MS line spectra (50-1300 *m*/*z*) were recorded at 8 Hz. The MS parameter settings were: end plate offset 500 V, capillary voltage 3000 V, nebuliser (N_2) pressure 3 bar, dry gas (N_2) flow and temperature 12 L min^{-1} at 275 °C, quadrupole ion energy 4 eV, low mass 90 *m*/*z*, collision energy in MS mode 7 eV, transfer time 75 µs, pre-pulse storage 6 µs. Nitrogen was chosen as collision gas in Auto-MS/MS mode to obtain fragments of the most intense ions, using a ramping of collision energies and isolation widths with increasing *m*/*z*. For recalibration, a sodium formate calibration solution was introduced into the ESI source before each sample. Next to the insect and plant samples, twelve blanks were extracted and measured using the identical method.

In Compass DataAnalysis 4.4 (Bruker Daltonics), for each sample the *m*/*z* axis was recalibrated using the sodium formate calibration peak. Then, the Find Molecular Feature algorithm was used for picking of molecular features (each defined by a retention time and

m/*z*), applying spectral background subtraction. Settings used for the algorithm were signal-to-noise threshold: 3, correlation coefficient threshold: 0.75, minimum compounds length: 16 spectra, smoothing width: 5. Molecular features probably belonging to the same metabolite were sorted together in so-called buckets, allowing the ion types $[M-H]^-$, $[M-H_2O-H]^-$, $[M+Cl]^-$, $[M+HCOOH-H]^-$, $[M+CH_3COOH-H]^-$, $[2M-H]^-$, $[2M+HCOOH-H]^-$, $[2M+CH_3COOH-H]^-$ and $[3M-H]^-$. For alignments of buckets across the set of samples, shifts of 0.1 min (retention time) and 5 mDa (*m*/*z*) were allowed in the processing software Compass ProfileAnalysis 2.3 (Bruker Daltonics). Within each bucket, the intensity (peak height) of the most intense molecular feature was used for quantification and divided by the height of the $[M+HCOOH-H]^-$ ion of hydrocortisone in the same sample. In the next step, fold changes were calculated for these features for each glucosinolate treatment as mean intensities in insect samples from the glucosinolate treatment divided by the mean intensities in insect samples from the solvent control treatment.

Supplement 5B: ***Details of myrosinase activity measurements.*** To measure myrosinase activities in insect samples, samples were extracted by maceration on ice in extraction buffer (200 mM Tris, 10 mM ethylenediaminetetraacetic acid, pH 5.5), centrifuged and supernatants were purified from glucosinolates via anion exchange columns as described in Travers-Martin et al. [1]. Myrosinase activities of extracts were measured in 96-well microplates after addition of glucosinolate substrates (1 mM benzyl glucosinolate, 4-hydroxybenzyl glucosinolate or 2-propenyl glucosinolate) dissolved in phosphate buffer or with the phosphate buffer only as control, and a colour reagent mixture consisting of glucose oxidase, peroxidase, 4-aminoantipyrine (Sigma Aldrich) and phenol (Merck, Darmstadt, Germany). The body samples were measured with all above-mentioned glucosinolate substrates, while the gut samples were only measured with the substrate 2-propenyl glucosinolate due to the small sample material. Absorptions were measured on a microplate photometer (Multiskan EX, Thermo Electron Corporation, Shanghai, China) at 492 nm for 45 min at room temperature against a glucose calibration curve. Furthermore, soluble protein concentrations

of the samples were measured according to Bradford [2] and used to express myrosinase activities.

References

1 Travers-Martin, N., Kuhlmann, F. & Müller, C. Revised determination of free and complexed myrosinase activities in plant extracts. *Plant Physiol. Biochem.* **46**, 506-516, doi:10.1016/j.plaphy.2008.02.008 (2008).

2 Bradford, M. M. A rapid and sensitive method for the quantification of microgram quantities of protein utilizing the principle of protein-dye binding. *Analytical Biochemistry* **72**, 248-254 (1976).
